# Determinants of health-related quality of life in older people with chronic musculoskeletal pain: a cross-sectional study

**DOI:** 10.1186/s12877-024-04669-z

**Published:** 2024-01-31

**Authors:** Hugo Pak-Yiu Fong, Shirley Yue-Kwan Choi, Maria Kwan-Wa Leung, Hermione Hin-Man Lo, Bo Wang, Samuel Yeung-Shan Wong, Regina Wing-Shan Sit

**Affiliations:** 1 The Jockey Club School of Public Health and Primary Care, Faculty of Medicine, The Chinese University of Hong Kong, New Territory, Hong Kong; 2 Department of Family Medicine, New Territories East Cluster, Hospital Authority, New Territory, Hong Kong; 3 The Nethersole School of Nursing, Faculty of Medicine, The Chinese University of Hong Kong, New Territory, Hong Kong

**Keywords:** Health-related quality of life, Older people, Chronic Musculoskeletal Pain

## Abstract

**Background:**

This study aimed to identify the significant physical, psychological, and social determinants associated with EuroQuol-5D (EQ-5D) among Chinese older people with chronic musculoskeletal pain, and to evaluate how these determinants affected the five dimensions of EQ-5D.

**Method:**

This is a cross-sectional study. Data were collected through a cohort involving 946 community-dwelling older people aged ≥ 60 with chronic musculoskeletal pain in Hong Kong. Selected independent variables were categorized into physical, psychological, and social domains. Physical variables included age, sex, body mass index (BMI), pain severity score, number of pain regions, the most painful site, and the number of comorbidities. Psychological variables included depression level measured using the 9-question Patient Health Questionnaire (PHQ-9), and anxiety level measured using the Generalized Anxiety Disorder Assessment (GAD-7). Social variables included living, marital, and social welfare recipient’s status. The dependent variables comprised the index scores and the five dimensions of the EQ-5D descriptive system. Ordinal least squares (OLS) model and logistic regression model were used for data analysis.

**Results:**

The mean age of the participants was 67.1 (SD = 5.1), with 77.6% being female. Higher pain severity scores (beta (β) coefficient =-0.044, *P* < 0.001), depression scores (β=-0.007, *P* < 0.001) and higher anxiety scores (β=-0.01, *P* < 0.001) were associated with lower EQ-5D index scores. Specifically, knee pain (β=-0.061, *P* < 0.001) was significantly associated with lower EQ-5D index scores. Participants with higher pain severity and depression scores were more likely to report problems in most EQ-5D dimensions. Participants with anxiety primarily faced challenges related to mood, and those with knee pain were more likely to have problems with mobility and daily activities.

**Conclusion:**

Among the selected determinants in our study, pain intensity, depression, anxiety, and knee pain were identified as key determinants associated with reduced HRQoL in older Chinese people with chronic musculoskeletal pain. Each of these determinants showed distinct associations with different dimensions of the EQ-5D, potentially informed resource allocation and the development of targeted interventions to improve the overall HRQoL of this specific population.

**Supplementary Information:**

The online version contains supplementary material available at 10.1186/s12877-024-04669-z.

## Background

Chronic musculoskeletal pain is a highly prevalent condition that significantly impacts individuals worldwide, resulting in significant physical, psychological, and social impairment [1, 2]. According to Global Burden of Disease (GBD) 2019, musculoskeletal pain conditions affect approximately 1.71 billion people globally, ranking high as one of the leading causes of disability worldwide [3]. Chronic musculoskeletal pain is particularly common among older adults, with prevalence rates ranging from 40–60%[4]. According to the World Health Organization report, musculoskeletal health conditions represent a global threat to healthy aging, with considerable negative effects on the older population [5]. 

Health-related quality of life (HRQoL) is a crucial indicator of the overall well-being and satisfaction experienced by individuals with a disease. It offers valuable insights into how the disease impacts various aspects of their daily lives, encompassing physical functioning, mental health, social interactions, and overall life contentment [6]. Considering HRQoL empowers healthcare providers to deliver more personalized, patient-centered care that addresses specific areas of impairment and enhances the overall quality of life for patients [7]. Moreover, it assists healthcare professionals in making informed treatment decisions that not only target disease symptoms, but also optimize patients’ overall well-being and functioning [8]. Additionally, by assessing HRQoL, policymakers and healthcare systems can effectively allocate resources to interventions with the highest potential for improving HRQoL outcomes [9]. 

Previous studies have extensively explored determinants impacting HRQoL among older individuals with various chronic diseases. For instance, research focusing on elderly hypertensive patients in China revealed that factors such as gender, age, income, and coexisting diseases significantly influenced their HRQoL. Similarly, a study on diabetes mellitus patients in India identified age, rural background, and symptoms of depression as key predictors of poorer quality of life, emphasizing the need for comprehensive and collaborative care [10, 11]. Despite the high prevalence of chronic musculoskeletal pain in older people and its established association with significantly lower HRQoL, [12–14] a notable research gap persisted in identifying key determinants of HRQoL in this demographic. Moreover, there was a lack of detailed understanding regarding the extent to which each determinant influences HRQoL in these individuals. Our study aimed to address this gap by examining the physical, psychological, and social determinants of HRQoL in older Chinese people with chronic musculoskeletal pain, and evaluating how these determinants affect the individual dimensions represented in HRQoL assessments. By identifying the most influential determinants of HRQoL in older individuals with chronic musculoskeletal pain, our study guides a strategic allocation of healthcare resources. It enables healthcare policymakers and practitioners to prioritize interventions and resources effectively, focusing on the areas with the most significant impact on HRQoL. Furthermore, by quantifying the impact of each determinant on the individual dimensions of HRQoL, we facilitate the creation of dimension-specific strategies to meet the needs of older adults with chronic musculoskeletal pain. This targeted intervention design enables us to effectively address specific aspects of HRQoL, thereby improving their well-being.

## Methods

### Study design

This cross-sectional study was conducted as part of the “CUHK-Jockey Club Pain Relief Project for Seniors,” a community-based charity program aimed at providing comprehensive non-pharmacological interventions to older adults suffering from chronic musculoskeletal pain. Data were collected from a cohort database established on January 1, 2019, which aimed to gather baseline health profiles of participants, monitor the longitudinal effects of pain on physical and psychosocial outcomes, and evaluate the potential impact of pain on healthcare utilization, quality of life, and mortality. The representativeness of the sample had been tested by measuring the differences between the sociodemographic of the present sample against the population aged ≥ 60 years reported in the Hong Kong census statistics 2022. (Appendix 1)

### Participants

The inclusion criteria included participants aged 60 years old or above; presented with chronic musculoskeletal pain, defined as regional pain (joints, limbs, back, neck), with degenerative conditions such as osteoarthritis, or musculoskeletal complaints that fall under the “chronic primary pain” classification of the International Classification of Disease-11 (ICD-11) [15]; and with pain that persisted or recurred ≥ 3 months [16]. Participants with cancer-related musculoskeletal pain were excluded; otherwise, no other specific exclusion criteria were adopted, although participants should have the ability to walk to the survey site, report data, and understand and sign an informed consent form.

### Settings

The study recruited participants from the Hong Kong Special Administrative Region (HKSAR) using a convenience sampling method. Participants were recruited through various community channels, including press conferences, television and radio programs, social media, digital platforms (such as websites and Facebook), and referrals from public and private primary care clinics. Data collection for the study was conducted through a two-step process. Initially, trained research assistants performed phone screenings to identify eligible participants. After that, individuals who met the study criteria were invited to provide written informed consent and participate in detailed face-to-face assessments. These assessments took place at a community-based interdisciplinary pain service center, led by experienced primary care providers.

### Independent variables

The biopsychosocial model, first proposed by George Engel in 1977, marked a significant shift from the traditional biomedical model by emphasizing the integration of biological, psychological, and social factors in understanding health and illness [17]. The model was subsequently adapted and refined by researchers such as Gatchel et al. for application in the context of pain, providing a comprehensive framework for pain assessment and management [18]. The biopsychosocial model of pain suggested that the perception of pain arose from the dynamic interplay among biological, psychological, and social factors. The model emphasized how the interaction among these factors influenced the patient’s individualized pain experience, potentially leading to a decline in quality of life [19, 20].

In our analysis, determinants were selected and categorized in accordance with the biopsychosocial model of pain, ensuring a comprehensive evaluation of their impact. The selection was further guided by existing research studies on quality of life determinants in chronic pain patients, [13, 21, 22] as well as the Core Outcome Domains for Chronic Pain Clinical Trials as recommended by IMMPACT [23].

The independent variables were grouped into the following domains:

- Physical domain: age, sex, body mass index (BMI), Brief Pain Inventory (BPI) severity score, [24, 25] number of pain regions, the most painful region (neck, shoulder, back, knee, foot, and ankle), and number of comorbidities.
- Psychological domain: depression level measured by the 9-question Patient Health Questionnaire (PHQ-9), [26] and anxiety level measured by the Generalized Anxiety Disorder Assessment (GAD-7) [27].
- Social domain: living arrangements (living alone or with family), marital status (single or married), and social welfare support status (whether they received community social security allowance).

### Dependent variable

The dependent variable was HRQoL, measured by the Hong Kong Chinese Version of the EuroQol-5 Dimension (EQ-5D) [28]. It consists of a descriptive system with five dimensions (mobility, self-care, usual activities, pain/discomfort, and anxiety/depression), each having five levels that capture the range of possible responses. The EQ-5D allows individuals to self-report their health status based on these dimensions and levels, and the results can be converted into a single health preference index, providing an overall assessment of HRQoL.

### Statistical analysis

Descriptive data were reported as percentages or means ± standard deviations. Ordinal least squares (OLS) model was used to study the relationship between different continuous and categorical independent variables with EQ-5D index score, and beta (β) coefficients were calculated. Binary multivariate logistic regression analysis was conducted to examine the odds ratios (OR) of participants reporting “having problems” in each dimension of the EQ-5D. Score of 1 in each dimension signifies “full health,” while a score greater than 1 indicates the individual is “reporting having problems” in that specific dimension. For the logistic regression analysis, a coding system was employed, where 0 represented reporting “full health,” and 1 represented reporting “having problems” in that dimension. Pairwise deletion was employed for missing values. Data analysis was conducted using R Statistical Software (version 4.1.3).

### Sample size calculation

The required sample size for our regression analysis was determined using the G*Power v3.1 statistical software [29]. In our regression model, encompassing fifteen predictors, we set an alpha level at 0.05, a conservative estimated effect size of 0.02, and a power of 0.90. Employing these parameters, an a priori power analysis conducted via G*Power software indicated a minimum sample size of 528 participants [30]. 

### Ethical consideration

The study was approved by relevant research ethics committee (CREC no. 2018.540). Written informed consent was obtained from all the participants.

## Results

A total of 946 participants were recruited with a mean age of 67.1 ± 5.1 years old and 77.6% were female. The mean BPI severity score was 4.2 ± 1.9; the mean number of painful regions was 3.4 ± 1.9, with the knee most commonly reported as the most painful region (39.2%). The mean scores for the PHQ-9 and GAD-7 were 6.3 ± 4.9 and 6.1 ± 5.3, respectively. The mean EQ-5D index was 0.70 ± 0.21. Baseline characteristics are summarized in Table 1.

**Table 1:** Baseline characteristics of participants (*N* = 946) *

		Mean/ Number	SD/ %
**Age (years)**		67.1	5.1
**Gender**	**Male**	221	22.4
	**Female**	767	77.6
**BMI**		23.5	3.8
**Number of pain sites**		3.4	1.9
**Number of comorbidities**		2.9	1.7
**BPI severity score**		4.2	1.9
**Most painful site**	**Ankle and foot**	80	8.1
	**Back**	323	32.7
	**Knee**	387	39.2
	**Neck**	48	4.8
	**Shoulder**	150	15.2
**PHQ-9**		6.3	4.9
**GAD-7**		6.1	5.3
**Marital status**	**Married**	650	68.7
	**Single**	296	31.3
**Living environment**	**Live with family**	832	84.2
	**Living alone**	156	15.8
**Social allowance**	**Yes**	334	33.8
	**No**	654	66.2

* Abbreviations: BMI, body mass index; BPI, Brief Pain Inventory; GAD-7, Generalized Anxiety Disorder Assessment; PHQ-9, 9-question Patient Health Questionnaire

The associations between individual physical, psychological, and social determinants with the EQ-5D index are summarized in Table 2. In the physical domain, higher BPI severity scores (β = -0.044, *P* < 0.01) and reporting knee pain (β = -0.061, *P* < 0.001) were found to be significantly associated with lower EQ-5D index scores. In the psychological domain, higher scores on the PHQ-9 (β = -0.007, *P* < 0.001) and GAD-7 (β = -0.01, *P* < 0.001) were significantly associated with lower EQ-5D index scores. No significant variables in the social domain were found to be associated with EQ-5D index scores.

**Table 2:** Multiple linear regression by OLS of the effect of individual determinants on Eq. 5D index score †

Variables	β	95% CI	*p* value
Age	-0.001	[-0.003, 0.001]	0.4
Female ‡	0.011	[-0.016, 0.037]	0.425
BMI	-0.003	[-0.006, 0.000]	0.057
Number of pain sites	0	[-0.006, 0.006]	0.886
Number of comorbidities	0	[-0.006, 0.007]	0.9
BPI severity score	**-0.044**	**[-0.050, -0.038]**	< 0.001*
Ankle and foot ‡	-0.03	[-0.076, 0.017]	0.212
Back ‡	-0.027	[-0.060, 0.006]	0.109
Knee ‡	**-0.061**	**[-0.094, -0.029]**	< 0.001*
Neck ‡	-0.002	[-0.058, 0.054]	0.943
PHQ-9	**-0.007**	**[-0.011, -0.004]**	< 0.001*
GAD-7	**-0.01**	**[-0.013, -0.007]**	< 0.001*
Single ‡	-0.014	[-0.042, 0.014]	0.326
Living alone ‡	0.013	[-0.022, 0.048]	0.459
Allowance ‡	-0.004	[-0.030, 0.021]	0.745

* Statistically significance;

† Abbreviations: β, beta coefficient; BMI, body mass index; BPI, Brief Pain Inventory; 95% CI, 95% confidence interval; EQ-5D, EuroQol-5 Dimension; GAD-7, Generalized Anxiety Disorder Assessment; PHQ-9, 9-question Patient Health Questionnaire;

‡ Reference levels: Gender (Male), Most painful site (Shoulder), Marital status (Married), Living status (Live with family), Allowance status (No allowance)

The effects of individual determinants on the five dimensions of EQ-5D are summarized in Table 3. Individuals with higher BPI severity scores were more likely to report “having problems” in the dimensions of mobility (OR = 1.44, *p* < 0.001), self-care (OR 1.27, *P* < 0.001), usual activities (OR 1.34, *P* < 0.001) and pain (OR2.43, *P* < 0.001). Individuals with higher PHQ-9 were more likely to report “having problems” in the dimensions of mobility (1.08, *P* = 0.002), self-care (1.07, *P* = 0.015), usual activities (1.08, *P* < 0.001), and mood (OR 1.11, *P* < 0.001). Those with higher GAD-7 only reported “having problems” in the dimension of mood (OR 1.27, *P* < 0.001). Individuals with knee pain reported “having problems” in the dimensions of mobility (OR 6.64, *P* < 0.001) and usual activities (OR 1.91, *P* < 0.001).

**Table 3:** Binary multivariate logistic regression of the effect of individual determinants on the five dimensions of EQ-5D †

Variables	EQ-5D1 (mobility)			EQ-5D2 (self-care)			EQ-5D3 (usual activities)			EQ-5D4 (pain/discomfort)			EQ-5D5 (anxiety/ depression)		
	OR	95%CI	*p* value	OR	95%CI	*p* value	OR	95%CI	*p* value	OR	95%CI	*p* value	OR	95%CI	*p* value
Age	1.01	0.98,1.05	0.449	1.01	0.97,1.05	0.661	**0.96**	**0.93,1**	0.025*	**0.93**	**0.88,0.99**	0.021*	0.97	0.93,1	0.083
Female ‡	0.86	0.6,1.24	0.429	0.73	0.47,1.16	0.175	1.07	0.74,1.55	0.71	0.75	0.38,1.43	0.397	0.82	0.55,1.24	0.351
BMI	**1.05**	**1.01,1.09**	0.023*	1.01	0.96,1.06	0.627	1.01	0.98,1.05	0.504	1.02	0.95,1.11	0.544	0.99	0.95,1.04	0.753
Number of pain sites	0.99	0.91,1.08	0.802	1.05	0.95,1.16	0.311	1.02	0.94,1.11	0.589	1.06	0.89,1.27	0.503	**1.1**	**1,1.2**	0.043*
Number of comorbidities	0.94	0.86,1.03	0.198	0.96	0.86,1.07	0.459	0.95	0.87,1.04	0.24	0.96	0.8,1.15	0.636	**1.12**	**1.01,1.24**	0.033*
BPI severity score	**1.44**	**1.31,1.58**	< 0.001*	**1.27**	**1.14,1.41**	< 0.001*	**1.34**	**1.23,1.46**	< 0.001*	**2.43**	**1.98,3.05**	< 0.001*	0.97	0.88,1.07	0.526
Ankle and foot ‡	**5.5**	**2.86,10.8**	< 0.001*	**0.3**	**0.11,0.71**	0.009*	0.81	0.41,1.56	0.531	1.21	0.39,4.14	0.746	0.88	0.43,1.75	0.712
Back ‡	**2.78**	**1.74,4.49**	< 0.001*	**0.44**	**0.26,0.76**	0.003*	1.33	0.85,2.1	0.217	1.27	0.54,2.88	0.575	0.89	0.55,1.46	0.651
Knee ‡	**6.64**	**4.16,10.8**	< 0.001*	0.69	0.42,1.13	0.135	**1.91**	**1.24,2.99**	0.004*	1.39	0.6,3.06	0.428	0.77	0.47,1.24	0.279
Neck ‡	1.7	0.79,3.67	0.173	0.56	0.22,1.35	0.216	1.1	0.52,2.31	0.806	8.81E + 06	0,1.04E + 132	0.984	0.75	0.32,1.72	0.498
PHQ-9	**1.08**	**1.03,1.13**	0.002*	**1.07**	**1.01,1.13**	0.015*	**1.08**	**1.04,1.13**	< 0.001*	0.94	0.86,1.04	0.231	**1.11**	**1.05,1.17**	< 0.001*
GAD-7	1.04	1,1.09	0.058	0.99	0.94,1.04	0.642	1.01	0.97,1.05	0.53	1.09	1,1.2	0.05	**1.27**	**1.21,1.33**	< 0.001*
Single ‡	1.05	0.71,1.56	0.804	0.83	0.53,1.33	0.438	0.79	0.55,1.15	0.22	0.59	0.25,1.29	0.204	0.84	0.55,1.29	0.421
Living alone ‡	1.24	0.76,2.02	0.383	0.81	0.45,1.45	0.492	0.92	0.58,1.45	0.71	0.62	0.24,1.67	0.34	0.96	0.56,1.63	0.876
Allowance ‡	1.21	0.85,1.74	0.29	1.03	0.67,1.57	0.896	0.94	0.66,1.32	0.716	0.7	0.36,1.37	0.298	1.02	0.68,1.52	0.92

* Statistically significant;

† Abbreviations: BMI, body mass index; BPI, Brief Pain Inventory; 95% CI, 95% confidence interval; EQ-5D, EuroQol-5 Dimension; GAD-7, Generalized Anxiety Disorder Assessment; OR, odds ratio; PHQ-9, 9-question Patient Health Questionnaire;

‡ Reference levels: Gender (Male), Most painful site (Shoulder), Marital status (Married), Living status (Live with family), Allowance status (No allowance)

## Discussion

In summary, older Chinese individuals with chronic musculoskeletal pain have a lower HRQoL compared to the general population in Hong Kong. This is evident from the distinct EQ-5D index score of 0.70, in contrast to the higher score of 0.92 observed in the general population [31]. In the context of the biopsychosocial model of pain, our study found that physical factors such as pain intensity and knee pain, and psychological factors like depression and anxiety, were significantly associated with lower HRQoL.

We further investigated the impact of pain intensity, knee pain, depression, and anxiety on different dimensions of EQ-5D. Our findings revealed that individuals with higher BPI severity scores were more likely to report “having problems” in four out of five EQ-5D dimensions, namely “mobility,” “self-care,” “usual activities,” and “pain/discomfort.” Interestingly, our study did not find a significant association between pain intensity and the “anxiety/depression”. This aligns with a previous study conducted by Björn Gerdle et al., which suggested that although anxiety and depression symptoms often accompany chronic pain, the correlation between pain intensity and psychological distress levels is relatively weak [32]. This complexity can be attributed to the intricate relationship between pain intensity and psychological distress, which can be influenced by individual coping strategies, attention, cognition, and emotion regulation [33, 34].

Compared to other musculoskeletal regions, older individuals who reported knee pain as their most severe pain region showed a significant association with reduced HRQoL. This aligns with previous studies which showed that knee pain was significantly associated with lower HRQoL in older populations [35, 36]. It is worth noting that knee pain was only found to be associated with the “mobility” and “usual activity” dimensions of EQ-5D. This was consistent with a previous study which found that mobility limitation reduces HRQoL [37]. Therefore, we suggest that when developing treatment strategies for knee pain in older individuals, interventions and resources should prioritize to enhance mobility and ability to perform usual activities, thus potentially improve their overall HRQoL.

Depression and anxiety are well-known to be associated with reduced HRQoL among patients with chronic pain [38, 39]. In terms of their influence on the individual dimensions of EQ-5D, the PHQ-9 was found to be significantly associated with “mobility,” “self-care,” “usual activities,” and “anxiety/depression.” On the other hand, the GAD-7 was only associated with the “anxiety/depression” dimension. Again, we did not find any association between psychological distress and the “pain/discomfort” dimension. These findings hold important clinical implications. When encountering chronic pain patients with depression, it is essential to not only manage the depressive symptoms but also focus on enhancing their physical functioning and self-management skills in order to improve their overall HRQoL. On the other hand, for those with comorbid anxiety, treatment can be specifically targeted at dealing with anxiety in order to improve their overall HRQoL.

To the best of our knowledge, this is the first study to evaluate the individual contributions of physical, psychological, and social factors on HRQoL within a single cohort of older individuals with chronic musculoskeletal pain. There were several limitations in this study. Firstly, participants were recruited using convenience sampling, a non-random method that could introduce statistical errors in group selection and potentially affect the validity of the results. This approach also carried a risk of self-selection bias, as those who volunteered might possess characteristics not representative of the general population of older adults. Secondly, the recruitment focused exclusively on community-dwelling individuals, which may limit the generalizability of our results to older adults with limited mobility or those residing in nursing homes. Lastly, our analysis was limited to selected determinants. While we focused on selected determinants across biological, psychological, and social domains, certain factors such as cognitive function and chronic medication use, were not included in the analysis. Additionally, although we addressed several social factors, our analysis did not provide a comprehensive coverage of all aspects of individuals’ social backgrounds.

Our findings have several clinical implications. Firstly, based on the biopsychosocial model of pain, we identified key determinants of HRQoL in older people with chronic musculoskeletal pain, emphasizing the need to address pain intensity, knee pain, depression, and anxiety as priorities for resource allocation. Secondly, our evaluation of individual determinants on the five dimensions of EQ-5D informs the development of targeted treatment strategies to enhance overall HRQoL. For instance, reducing pain intensity should focus on improving mobility, self-care, and the ability to perform usual activities. Interventions targeting knee pain should prioritize improvements in mobility and self-care. Patients with comorbid depression and chronic pain would benefit from multidisciplinary care, while individuals with comorbid anxiety may benefit from targeted treatment addressing their anxiety symptoms.

## Conclusion

Among the selected determinants in our study, pain intensity, depression, anxiety, and knee pain were identified as key determinants associated with reduced HRQoL. Older people with higher pain intensity and greater depressive symptoms were more likely to encounter difficulties across various dimensions of the EQ-5D. Participants with anxiety primarily faced challenges related to mood, and those with knee pain were more likely to have problems with mobility and daily activities. These findings have the potential to guide the allocation of resources and the development of targeted interventions aimed at enhancing HRQoL.

### Electronic supplementary material

Below is the link to the electronic supplementary material.

Supplementary Material 1

## Data Availability

The datasets used and/or analysed during the current study are available from the corresponding author on reasonable request.

## Abbreviations

HRQoL Health: Related Quality of Life
EQ: 5D EuroQuol-5D
BMI Body: Mass index
BPI: Brief Pain Inventory
PHQ-9: 9-question Patient Health Questionnaire
GAD-7: Generalized Anxiety Disorder Assessment
GBD: Global Burden of Disease
ICD-11: International Classification of Disease-11
HKSAR: Hong Kong Special Administrative Region
OLS: Ordinal least squares
OLS: Ordinal least squares
β: Beta
OR: Odds ratios
CREC: Clinical Research Ethics Committee
