# Supplementary material for: Determinants of health-related quality of life in older people with chronic musculoskeletal pain: a cross-sectional study
Source: BMC Geriatr. 2024 Jan 31;24:119. doi: 10.1186/s12877-024-04669-z (PMC10832201; doi:10.1186/s12877-024-04669-z)
Supplement: Supplementary file 1 — Supplementary Material 1 [file 12877_2024_4669_MOESM1_ESM.docx]

Appendix 1: Comparisons of demographic characteristics in the cohort study and population estimates 2022 census in Hong Kong * †

|  | | Current sample (%) | Hong Kong 2021 census (%) | Effect size |
| --- | --- | --- | --- | --- |
| Gender | |  |  |  |
|  | Male | 22.3 | 48.6 | 0.012 |
|  | Female | 77.7 | 51.4 |  |
| Age | |  |  |  |
|  | 60-64 | 35.0 | 31.1 | 0.010 |
|  | 65-69 | 35.4 | 25.1 |  |
|  | 70-74 | 21.6 | 18.9 |  |
|  | 75-79 | 5.6 | 10.1 |  |
|  | 80-84 | 1.5 | 8.4 |  |
|  | 85-89 | 0.9 | 6.3 |  |
| Marital status | |  |  |  |
|  | Never married | 9.8 | 27.1 | 0.007 |
|  | Married | 68.6 | 55.7 |  |
|  | Widowed | 14.2 | 10.4 |  |
|  | Divorced | 7.5 | 6.7 |  |
| Living status | |  |  |  |
|  | Composed of couple | 32.4 | 17.2 | 0.008 |
|  | Composed of relationship combination | 51.8 | 60.1 |  |
|  | One-person households | 15.6 | 20.2 |  |
|  | Non-relative households | 0.2 | 2.5 |  |
| * The weighted data were used for analysis;  † The data from all age groups were used for marital status and living status in Hong Kong 2021 census, as the data for people over 60 were not available. | | | | |
